# Thrombospondin-1 as a Paradigm for the Development of Antiangiogenic Agents Endowed with Multiple Mechanisms of Action

**DOI:** 10.3390/ph3041241

**Published:** 2010-04-23

**Authors:** Marco Rusnati, Chiara Urbinati, Silvia Bonifacio, Marco Presta, Giulia Taraboletti

**Affiliations:** 1 Unit of General Pathology and Immunology, Department of Biomedical Sciences and Biotechnology, School of Medicine, University of Brescia, Brescia, 25123, Italy; E-Mails: urbinati@med.unibs.it (C.U.); presta@med.unibs.it (M.P.); 2 Tumor Angiogenesis Unit, Department of Oncology, Mario Negri Institute for Pharmacological Research, Bergamo, Italy; E-Mails: bonifacio@marionegri.it (S.B.); taraboletti@marionegri.it (G.T.)

**Keywords:** angiogenesis, tumor, integrins, interactions, thrombospondin-1

## Abstract

Uncontrolled neovascularization occurs in several angiogenesis-dependent diseases, including cancer. Neovascularization is tightly controlled by the balance between angiogenic growth factors and antiangiogenic agents. The various natural angiogenesis inhibitors identified so far affect neovascularization by different mechanisms of action. Thrombospondin-1 (TSP-1) is a matricellular modular glycoprotein that acts as a powerful endogenous inhibitor of angiogenesis. It acts both indirectly, by sequestering angiogenic growth factors and effectors in the extracellular environment, and directly, by inducing an antiangiogenic program in endothelial cells following engagement of specific receptors including CD36, CD47, integrins and proteoglycans (all involved in angiogenesis ). In view of its central, multifaceted role in angiogenesis, TSP-1 has served as a source of antiangiogenic tools, including TSP-1 fragments, synthetic peptides and peptidomimetics, gene therapy strategies, and agents that up-regulate TSP-1 expression. This review discusses TSP-1-based inhibitors of angiogenesis, their mechanisms of action and therapeutic potential, drawing our experience with angiogenic growth factor-interacting TSP-1 peptides, and the possibility of exploiting them to design novel antiangiogenic agents.

## 1. Neovascularization

**Figure 1:**
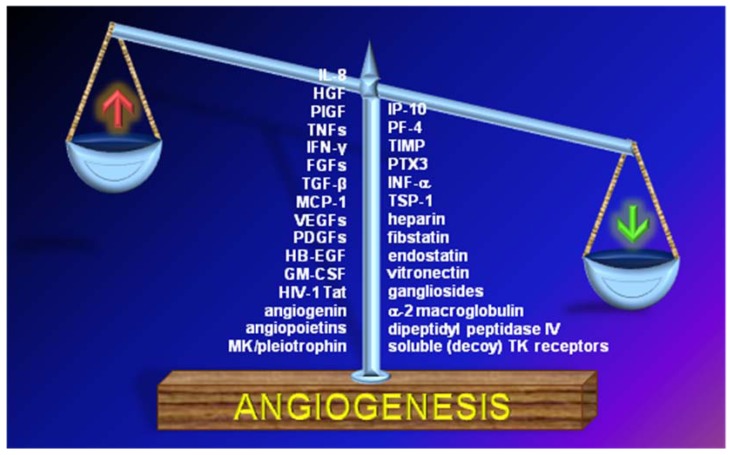
The balance between the production and release of pro- and antiangiogenic molecules regulates neovascularization. For a more exhaustive list of antiangiogenic compounds and abbreviations, see Table 1 and Table 2.

Angiogenesis is the process of new blood vessel formation from existing ones. It takes place in embryonic development and inflammation [1] and during angiogenesis-dependent diseases, including cancer [2]. In view of its essential contribution to physiological processes and major pathologies, angiogenesis is tightly controlled, mainly through the balance between the production and release of pro-angiogenic and antiangiogenic molecules (Figure 1).

Pro-angiogenic molecules are a heterogeneous group of proteins that include the vascular endothelial growth factors (VEGFs) and fibroblast growth factors (FGFs). They induce angiogenesis by interacting with tyrosine kinase receptors (TKRs) expressed on the endothelial cell (EC) surface. A common theme among angiogenic growth factors (AGFs) is their interaction with (co)receptors other than TKRs (e.g. heparan sulfate proteoglycans (HSPGs) and integrins [3]). This complex pattern of extracellular interactions is mirrored by the intricacy of the signal transduction pathway(s) triggered by AGFs in ECs [4]. Once stimulated, ECs acquire the “angiogenic phenotype”, namely the ability to execute all the different steps of the angiogenic process, including extracellular matrix (ECM) degradation, a change of surface expression of adhesion molecules, proliferation, and chemotactic migration [4].

## 2. Antiangiogenic Compounds

Antiangiogenic compounds are a heterogeneous group of molecules that includes proteins, polysaccharides and glycosphingolipids found in the body fluids and ECM. A common theme among antiangiogenic compounds is their ability to directly bind and sequester AGFs in the extracellular environment, thus hampering their interaction with ECs (Table 1). 

**Table 1:** Endogenous antiangiogenic molecules that bind and sequester AGFs in the extracellular environment. TSP-1 is highlighted in grey.

Molecule	AGF bound	Reference
TSP-1	FGF2, VEGF, HGF, HIV-1 Tat, TGF-β_1_	[5,6,7,8], [9,10], [11], [12], [13]
α_2_-macroglobulin	FGF2, VEGF, TGF-β, IL8, TNF	[14], [15], [16], [17], [18]
heparin	FGF2, VEGF, HIV-1 Tat, HGF	[19], [20], [21], [22]
pentraxin-3 (PTX3)	FGF2, FGF8	[23]
factor VII-activating protease	FGF2, PDGF	[24], [25]
platelet factor 4 (PF-4)	FGF2, VEGF	[26], [27]
SPARC	VEGF, PDGF	[28], [29]
CXCL13	FGF2	[30]
gangliosides	FGF2	[31]
fibstatin (fibronectin fragment)	FGF2	[32]
vitronectin	FGF2	[33]
soluble VEGF receptor (VEGFR)-1	VEGF	[34]
ADAMTS1	VEGF	[35]
heparin affin regulatory peptide (HARP)	VEGF	[36]
connective tissue growth factor (CTGF)	VEGF	[37]
soluble endoglin	TGF-β_1_	[38]
decorin	TGF-β_1_	[39]
secretory component	IL8	[40]

However, under certain conditions, the direct interaction with a given ligand may lead to oligomerization of the AGF, its protection from proteolytic degradation and a rise in local concentration. This, in turn, will lead to proangiogenic rather than antiangiogenic effects, as already demonstrated for AGF binding to heparin, collagen and fibrinogen/fibrin (see [3] and references therein).

**Figure 2:**
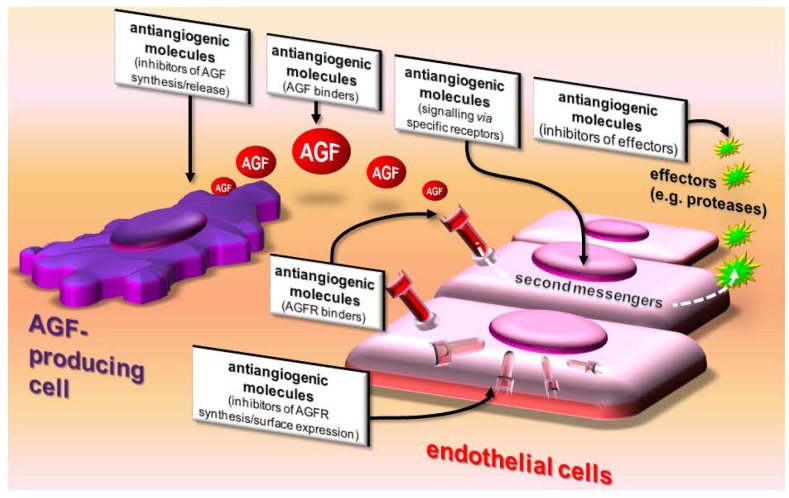
Action on angiogenesis. Antiangiogenic molecules affect AGFs by acting on AGF-producing cells, AGFs themselves, AGF receptors (AGFR), ECs, and angiogenesis effectors produced by activated ECs.

Besides binding AGFs, antiangiogenic molecules can inhibit angiogenesis by: i) inhibiting AGF production by tumor cells; ii) inhibiting surface expression of AGF-receptors on ECs; iii) binding (and masking) AGF receptors; iv) reducing EC responsiveness to AGFs (usually by engaging specific receptors that modify the EC phenotype); v) inhibiting effectors of angiogenesis produced by ECs (*i.e.,* proteases required for ECM degradation) (Figure 2 and Table 2).

Two main considerations emerge from Table 1 and Table 2: i) some antiangiogenic molecules (including TSP-1) target different AGFs simultaneously; ii) some antiangiogenic molecules (including TSP-1) inhibit the angiogenic process through multiple mechanisms. These indications may provide useful suggestions for designing therapeutic strategies to inhibit angiogenesis, since pathological neovascularization is often the result of the simultaneous, non-redundant actions of various AGFs [41,42]. Inhibiting neovascularization by drugs directed against a single AGF/TKR presents several limitations [41]. Moreover, developing drugs acting on multiple targets/mechanisms may limit the insurgence of drug resistance, which is a major problem with conventional antineoplastic therapies.

TSP-1 interferes simultaneously with several AGFs (Table 1) through different mechanisms of action (Table 2), thus offering a paradigm for the development of antiangiogenic drugs. This review discusses TSP-1 and TSP-1-based inhibitors of angiogenesis, their mechanisms of action and therapeutic potential.

## 3. Structure and Biological Activity of TSP

The mammalian family of TSPs comprises five members, including TSP-1 and TSP-2, which form group A, homotrimeric TSPs. They are very similar in structure and can all inhibit angiogenesis, although they are expressed differently in various tissues during development and adulthood. 

**Table 2:** Endogenous antiangiogenic molecules and their mechanisms of action. TSP-1 is evidenced in grey.

MOLECULE	MECHANISM OF ACTION
**inhibition of AGF expression/release by producing cells**	
homocysteine	lowering FGF2 levels [43]
interleukin (IL)-12	lowering FGF2 levels [44]
**TSP-1**	lowering FGF2 levels [45]
**inhibition/interference with AGF receptors on ECs**	
IL-1, IFN-γ	TK- FGF receptors (TK-FGFR) down-regulation [46]
anosmin-1	TK-FGFR occupancy [47]
thromboxane	inhibition of TK-FGFR1 internalization [48]
soluble form of TKR	formation of heterodimers with TK-FGFR1 [49]
antithrombin	HSPG down-regulation [50]
PF4	HSPG occupancy [51], unknown [52]
**MOLECULE**	**MECHANISM OF ACTION**
endostatin	HSPG occupancy [53]
kallistatin	HSPG occupancy [54]
histidine-rich glycoprotein	HSPG occupancy [51]
endosulfatases	HSPG desulfation [55,56]
heparinase	HSPG degradation [57]
**TSP-1**	HSPG occupancy [9], integrin occupancy [48]
**inhibition/interference with AGF-activated second messengers in ECs**	
heat-shock proteins 70 and 90	down-regulation of pAkt, c-Raf-1 and ERK_1/2 _[58]
sprouty proteins	inhibition of TK-FGFR signalling [59]
homeobox gene GAX	inhibition of NF-kB signalling [60]
semaphorin-3F	inhibition of ERK_1/2_ signalling [61]
angiostatin [a plasminogen (Plg) fragment]	inhibition of ERK_1/2_ signalling [62]
ghrelin	inhibition of TKR/MAPK signalling [63]
lysophosphatidylcholine	inhibition of ras/ERK_1/2_ signalling [64]
pigment epithelium-derived factor	inhibition of Fyn signalling [65]
**TSP-1**	inhibition of VEGF-mediated Akt signalling [66]
**modification of EC apoptosis, phenotype, responsiveness to AGFs**	
cleaved HMW kininogen	tropomyosin engagement [67]
IL-4	alteration of cell cycle [68]
kininostatin (kininogen fragment)	inhibition of cyclin D1 expression [69]
vitamin D3-binding protein	CD36 engagement [70]
endostatin	Shb activation [71]
histidine-rich glycoprotein	tropomyosin engagement [72]
endostatin	cytoskeleton organization [73], Shb activation [71]
**TSP-1**	TNF-α over-expression [74], CD36 engagement [66,75], apoptosis [45,66,74], ECM modification [76]
**inhibition/interference with angiogenesis effectors**	
IL-12	inhibition of FGF-induced proteases [44]
tissue inhibitor metalloproteinase (TIMP)-2, 4	inhibition of FGF-induced proteases [77]
kallistatin	inhibition of FGF-induced proteases [54]
**TSP-1**	inhibition of FGF-induced proteases [78], binding to matrix metalloproteinase-2 (MMP-2) [79]
**unknown mechanism of action**	
collagen I [80], alphastatin (fibrinogen fragment) [81], CXCL14 [82], IL-12 [83], IP-10 [84], vasostatin [85], vasculostatin (fragment of brain angiogenesis inhibitor-1) [86], TGF-β1 [87], TNFs [88], somatostatin [89], retinoids [90], apolipoprotein(a) [91], prolactin (16 kDa fragment) [92]	

In view of the extensive literature, here we only report inhibitors of FGF2 as a prototypic AGF.

**Figure 3:**
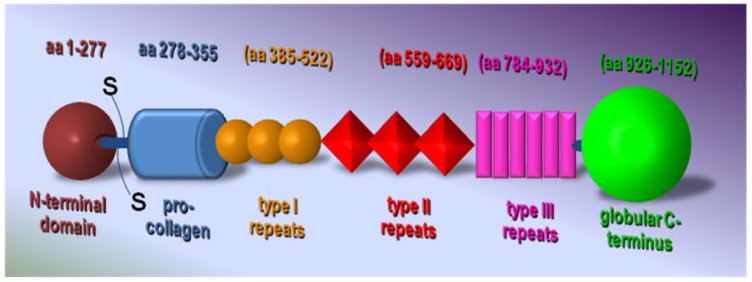
Schematic representation of TSP-1 structure.

TSP-1 was the first endogenous inhibitor of angiogenesis to be identified [30,93]. Each TSP-1 monomer is formed by an N-terminal globular domain, followed by the coiled-coil oligomerization domain, a von Willebrand Factor type C procollagen domain, three properdin-like type I repeats, two epidermal growth factor-like type II repeats and a signature domain comprising a third type II repeat, the calcium-binding wire-type III repeats, and the lectin-like C-terminal globular domain [14] (Figure 3).

Type I repeats is a relatively small region commonly considered the main antiangiogenic site of TSP-1. Interestingly, this domain is also present in several other proteins, often giving them significant antiangiogenic activity [94,95].

The region comprising the third type II repeats, the type III repeats and the C-terminal globular end is the most conserved region in the TSP family [96]. Its properties are affected by calcium. The cooperative binding of calcium ions is the main feature of type III repeats and profoundly affects the structure/availability of active sequences in the whole cassette. The type III repeats contain a cryptic integrin recognition motif RGD [97], two sequences that bind cathepsin G and neutrophil elastase [98], and binding sites for collagen V and FGF2. These binding sites become exposed only after drastic structural changes induced by a low calcium concentration or disulfide bond reduction, illustrating the importance of environmental conditions for the bioavailability and activity of these sequences [7,97,98,99]. TSP-1 is active both as a whole molecule and as fragments [100], a property shared by the matricellular PTX3 [23,101], whereas most endogenous angiogenesis inhibitors are fragments of larger molecules with no intrinsic antiangiogenic activity (such as fibronectin, kininogen and plasminogen (Table 1 and Table 2).

**Figure 4:**
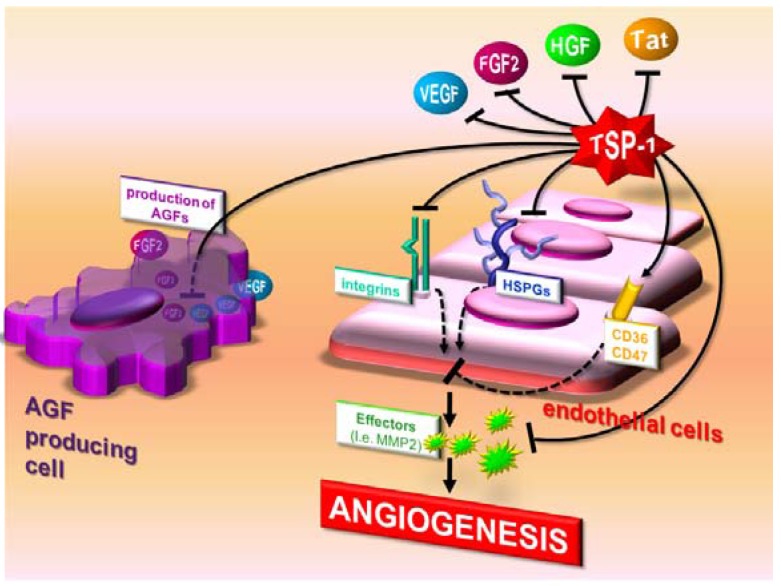
Direct and indirect antiangiogenic actions of TSP-1. TSP-1 sequesters AGFs in the extracellular environment and masks various AGF receptors. TSP-1 also reduces EC responsiveness to AGFs and induces apoptosis by activating CD36. It binds matrix metalloproteinase-2 (MMP-2), favoring its clearance. Finally, it inhibits AGF production by tumor cells.

TSP-1 has an extremely complex, context-dependent effect on angiogenesis, reflecting the heterogeneity of its functional domains, each interacting with selected cell receptors, AGFs, ECM components, and proteases [100]. Thus, in a given biological setting, the local/temporal expression of these ligands and the availability of each TSP-1 domain drive the pattern of molecular interactions which, in turn, dictate the biological effects of TSP-1 [78]. As a consequence, TSP-1 exerts both pro- and antiangiogenic effects *in vitro* and *in vivo* depending on its concentration [102], free or ECM-associated status [103,104,105] and oligomerization [106,107,108].

TSP-1 affects angiogenesis both directly and indirectly. As a direct inhibitor, it interacts with specific receptors (*i.e.,* CD36 and CD47 [109]) on ECs to affect apoptosis and functions related to angiogenesis. As an indirect inhibitor, it binds to and influences the activity/bioavailability of various mediators of angiogenesis, including AGFs, cytokines and proteases (Table 1 and Table 2, Figure 4).

### 3.1. Direct effects of TSP-1

TSP directly affects ECs and tumor cells by interacting with specific receptors. CD36 was the first TSP-1 receptor identified [110]. It is an 88-kDa glycoprotein expressed by many cell types including ECs [111]. TSP-1 and CD36 interact through the CLESH-1 domain in CD36 and the type I repeats in TSP-1 [112]. CD36 constitutively associates with β1 integrins and VEGF receptor 2 (VEGFR-2) in ECs [113,114], with interesting implications for the cross-talk among TSP-1, CD36, VEGFR-2 and integrins in the antiangiogenic action of TSP-1. This interaction has manifold consequences: it inhibits FGF2-induced EC migration, morphological organization [112,113], production of nitric oxide (NO) [115] and angiogenesis *in vivo* [116], induces apoptosis in ECs [116] and tumor cells [117,118] and down-regulates the expression and phosphorylation of VEGFR-2 on EC surface [113,114].

CD47 was originally identified as the receptor that mediates cell adhesion and spreading to TSP-1 [119,120]. CD47 also forms a signalling complex with integrins [121]. By binding to CD47, TSP-1 inhibits NO-cGMP signalling, hence also neovascularization [115,122].

TSP-1 binds heparin and HSPGs (syndecan-1 and 4, perlecan, decorin) through its N-terminal domain (Table 3). This interaction can have antiangiogenic effects. TSP-1 displaces VEGF from EC HSPGs, inhibiting angiogenesis [9]. However, by binding to syndecan-4, TSP-1 can also exert pro-angiogenic effects, protecting ECs from apoptosis and stimulating tubulogenesis [123]. The heparin binding motif Hep II also comprises the binding sequence for α6 integrin, pointing to cooperation between HSPGs and integrins, as already demonstrated for CD36 and CD47 (see above).

The low-density lipoprotein receptor-related protein (LRP) acts as a TSP-1 receptor. Like HSPGs, LRP can exert opposite effects on angiogenesis. It mediates endocytosis of the TSP-1/VEGF [124] and TSP-1/MMP-2 [79] complexes, contributing to the CD36-independent inhibition of VEGF angiogenic activity by TSP-1. However, the binding of TSP-1 to calreticulin (Table 3) enhances calreticulin binding to LRP that, in turn, induces EC motility and focal adhesion disassembly [125,126].

TSP interacts with several β1 integrins and with α_v_β_3_, involved in angiogenesis. Different binding sites for integrins have been mapped in TSP-1 (Table 3). Again, the various interactions between the different functional domains of TSP-1 and the different integrins can mediate both pro- [127] and antiangiogenic [128] effects. However, preclinical studies indicate that small TSP-1 peptides containing the integrin binding sites, as well as disintegrins or anti-integrin antibodies, block EC pro-angiogenic functions such as adhesion, proliferation, survival, wound healing, motility and angiogenesis *in vivo* [127,129,130].

### 3.2. Indirect effects of TSP-1

Besides cell surface receptors, TSP-1 interacts with several other partners, including AGFs (Table 3). TSP-1 binds FGF2 with affinity similar to the FGF2/HSPG interaction. Accordingly, heparin prevents the TSP-1/FGF2 interaction and TSP-1 prevents the FGF2/HSPG interaction [5]. As a consequence of its interaction with FGF2, TSP-1 inhibits FGF2-triggered proliferation and chemotaxis in ECs [5,6,7]. Finally, TSP-1 prevents FGF2 accumulation in the ECM, favoring its mobilization as inactive TSP-1/FGF2 complexes [6]. These observations suggest that free TSP-1 acts as a scavenger for ECM-associated FGF2, affecting its location, bioavailability and function.

TSP-1 binds both free and cell-associated VEGF [9], suggesting that it regulates the bioavailability of VEGF in the microenvironment and its capacity to bind to its EC receptors during neovascularization. Also, TSP/VEGF complexes are internalized *via* LRP-1 [131]. This contributes to TSP-1’s ability to inhibit VEGF-induced EC tubulogenesis *in vitro* and angiogenesis *in vivo* [9].

**Table 3:** TSP-1 ligands and their binding domains in the TSP-1 structure.

Ligand			Binding domain in TSP-1	Reference
free molecules (body fluids)	AGFs	FGF2	• type III repeats	[7]
		VEGF	• type I repeats	[37]
		HGF	• 3D conformation	[11]
		HIV-1 Tat	N.D.	[12]
		TGF-β	• 2nd type I repeats (RFK sequence)	[132,133,134]
			• type I repeats (WSXW sequence)	[133,134]
		PDGF-BB	• 3D conformation	[135]
	proteases and regulators	MMP-2	• type I repeats	[136]
		Plg/plasmin	N.D.	[137,138,139]
		tissue Plg activator	N.D.	[140]
		urokinase Plg activator	N.D.	[141]
		neutrophil elastase	• type III repeats	[142]
		cathepsin G	• type III repeats	[142,143]
		tissue factor inhibitor	N.D.	[144]
	others	heparin	• N-ter domain [motifs Hep I (aa 17-35) & Hep II (aa 78-94)]	[103,145]
			• type I repeats	[146,147]
			• signature domain	[148]
		histidine-rich glycoprotein	N.D.	[149]
		factor V	N.D.	[150]
		angiocidin	• 2nd and 3rd type I repeats (CSVTCG sequence)	[151]
		calumenin	• N-ter domain (aa 21- 228)	[152]
		endostatin	N.D.	[153]
	cell surface receptors	CD36	• type I repeats	[112]
		CD47	• C-ter domain	[118,122,154]
		HSPGs	• N-ter domain [motifs Hep I (aa 17-35) & Hep II (aa 78-94)]	[103,145]
			• signature domain	[148]
		sulfated glycolipids	• N-ter domain	[155]
			• 3D conformation	[155]
		LRP	• N-ter domain	[126,155]
		VLDL receptor	• N-ter domain	[156,157]
		calreticulin	• N-ter domain (aa 17-35)	[126,158]
		integrins	• N-ter domain	[107,129,159,160,161]
			• type I repeats	[128,130,162]
			• type III repeats (RGD sequence)	[118,163]
	ECM	collagen I	N.D.	[164]
		collagen V	• procollagen domain + type I & II repeats	[165,166]
		fibronectin	• N-ter domain + type I & II repeats	[167,168]
		laminin	N.D.	[165]
		fibrinogen/fibrin	• N-ter domain	[157]
			• procollagen domain	[146]
			• type I repeats	[169,170]
		von Willebrand factor	• signature domain	[171]
		dermatan sulfate	• N-ter domain (KKTR sequence)	[172]
		chondroitin sulfate	• N-ter domain	[155]
		IGF-binding protein-5	• N.D.	[173]

N.D., not determined. HSPGs, reported here as cell surface receptors, are also constituents of ECM. Conversely, dermatan- and chondroitin-sulfates, reported as ECM components, also exist as saccharidic chains of cell surface proteoglycans.

TSP-1 binds HGF in a calcium-independent manner. Heat denaturation reduces its binding to HGF, suggesting that a proper 3D conformation is required [11]. Mature two-chain and precursor single-chain HGF both bind to TSP-1. Heparin prevents this interaction but does not disrupt established complexes. At a biological level, TSP-1 inhibits HGF-induced chemotaxis of ECs *in vitro* and HGF-induced angiogenesis *in vivo* [11].

TSP-1 binds HIV-1 Tat [12] inhibiting Tat-induced EC migration *in vitro* and angiogenesis *in vivo* [12,174]. It also binds and activates transforming growth factor (TGF)-β_1_ through sequences located in the type I repeats [132,133,134]. TSP-1-associated TGF-β_1 _is biologically active and protected from inactivation. As a result, the inhibition of ECs by TSP-1 is at least partly mediated by complexed TGF-β_1 _[13,134]. TSP-1 binds both free or substrate-associated PDGF-BB in a calcium-dependent way [135], but the biological importance of these interactions remains to be clarified.

As a matricellular protein, TSP-1 participates in organizing the ECM, which provides environmental and positional cues to ECs during angiogenesis. ECM components interact to form a complex structural framework, and TSP-1 binds several of them (Table 3), with various consequences on ECM assembly and adherent EC behavior. Also, ECM is continuously remodeled by proteases. TSP-1 binds several proteases, including MMP-2, plasmin, neutrophil elastase and cathepsin G [79,98,136,175,176]. It promotes MMP-2 clearance *via* endocytosis by LRP [79,175] and suppresses MMP-3-mediated activation of proMMP-9 [177]. Conversely, it stimulates MMP-9 expression in ECs, promoting tubulogenesis [178]. The antiangiogenic 140 kDa TSP-1 fragment induces TIMP-2 over-expression [103], whereas the proangiogenic N-terminal domain of TSP-1 increases MMP-9 and MMP-2 release and reduces TIMP-2 expression by ECs.

**Figure 5:**
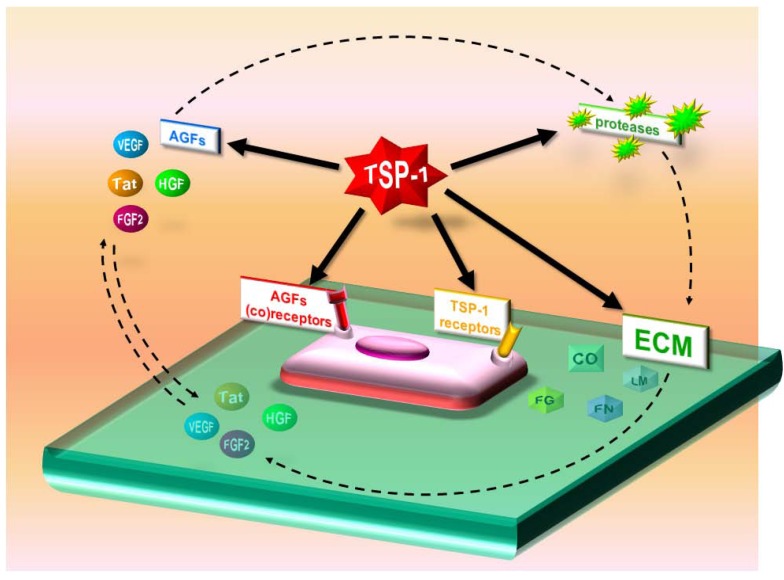
TSP-1 interactome. AGFs bind receptors inducing proteases that remodel ECM and mobilize AGFs, creating an environment favorable to EC proliferation and migration. TSP-1 binds several of these regulators, orchestrating their interactions/activities and leading to fine tuning of EC behavior during neovascularization.

Thus many of TSP-1’s effects on neovascularization are due to its ability to bind several molecules present in body fluids, ECMs, and the EC surface. It can therefore be envisaged at the centre of a complex interplay among AGFs, ECM components and their receptors and proteases. Through these multiple interactions, TSP-1 orchestrates their bioavailability, mutual binding and activities, leading to regulation of EC behavior during angiogenesis (Figure 5).

The “multi-binding” properties of TSP-1 depend on its modular structure in which several binding sequences are in close proximity, a feature that may lead to the formation of large multi-molecular complexes [140,149] in which the activity of each sequence becomes context-dependent, according to the environmental conditions and the predominant ligand. The ”multi-binding” capacity may also favor the coupling of some of its receptors (e.g. CD36, CD47, integrins, HSPGs and VEGFR-2, see above).

## 4. Therapeutic Exploitation of TSP-1 as an Antiangiogenic Agent

With its multifaceted roles in the control of angiogenesis and oncogenesis, TSP-1 could be exploited therapeutically by different approaches.

### 4.1. TSP-1 upregulation

This is based on the observation that many antiangiogenic molecules act indirectly by upregulating the production of TSP-1. Thus, the simplest way to exploit TSP-1’s antiangiogenic potential would be to deliver molecules that induce its over-expression in producing cells (Table 4).

**Table 4:** Natural and synthetic molecules that induce over-expression of TSP-1.

Molecule	References
glucose	[179]
peroxisome proliferator-activated receptor agonist fenofibrate	[180]
trichostatin-A	[181]
retinoic acid	[182,183]
somatostatin receptor subtype 2	[10]
cyclic adenosine 5'-monophosphate-activated guanine nucleotide exchange factor for Rap1	[184]
angiostatin	[185]
PHA -665752 (a small molecule, ATP-competitive inhibitor of c-Met receptor)	[186]
delta4-tibolone	[187]
phorbol 12-myristate 13-acetate	[183]
fibulin-5	[188]
angiotensin II and its agonist CGP42112A	[189,190]
endostatin	[191]
estradiol	[192]
progesterone and raloxifene	[193]
IL-6	[183]
IL-18	[194]
erythropoietin	[195]
epidermal growth factor	[196]
TFG-β1, FGF2	[197]
thrombin	[198]
inhibitors of DNA methyltransferases and histone deacetylases	[199]
CD26-processed chemokines CXCL12 and CCL5	[200]

The practical exploitation of this approach might be hampered by the unpredictable effects of non-selective over-expression of such a pleiotropic molecule. Nonetheless, it is interesting to note that the antiangiogenic, antineoplastic activity of metronomic, low-dose cyclophosphamide has been associated with increased levels of TSP-1 [201,202]. Similarly, TSP-1 is induced in colon cancer models after treatment with 5-FU [203], in rat prostate tumors treated with cyclophosphamide, doxorubicin or paclitaxel [204], in head and neck squamous carcinoma cells treated with docetaxel [205], in neuroblastoma cells treated with valproic acid [206], and in HT-29 colon cancer xenografts treated with metronomic irinotecan [207]. Metronomic irinotecan also raises plasma levels and gene expression of TSP-1 in patients with metastatic colorectal cancer [208].

### 4.2. Gene therapy

**Figure 6:**
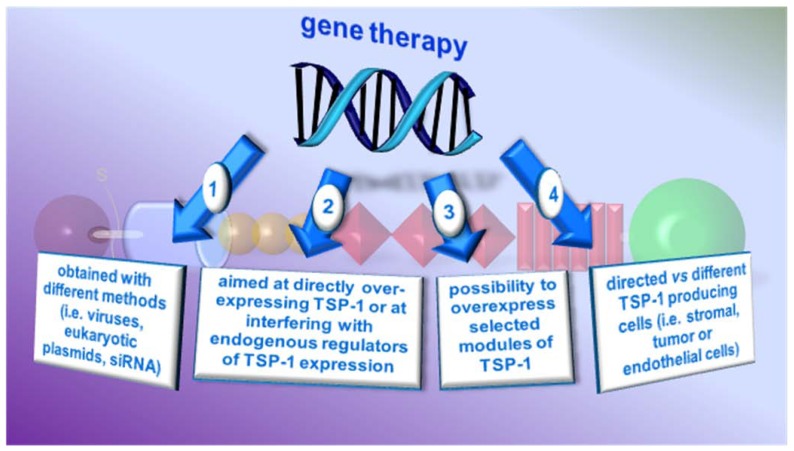
Advantages of TSP-1-based gene therapy. By different strategies (1) and by targeting different cell types (4), it is possible to induce directly the expression of the TSP-1 gene, to stimulate or inhibit the expression of TSP-1 enhancers/inhibitors (2) or to express selected TSP-1 modules (3).

More controlled TSP-1 over-expression could be achieved by gene therapy, whose advantages are schematized in Figure 6. TSP-1 over-expression can be obtained by targeting the TSP-1 gene itself or a number of oncogenes/oncosuppressor genes that influence its expression. Adams and co-workers [106] nicely demonstrated the versatility of the gene therapy approach by using different TSP-1 modules differing in their capacity to selectively interact with various ligands.

The list below describes some TSP-1-based gene therapy strategies:

i) Fibroblasts retrovirally transduced to produce high levels of TSP-1 resulted in high levels of the protein that inhibited angiogenesis and tumor growth in different models [209]; ii) recombinant adeno-associated virus (AAV)-mediated delivery of the three type I repeats (3TSR) resulted in expression of the transgene in normal tissues, reduced VEGF-induced angiogenesis, reduced tumor growth and microvessel density both locally and at distant sites [210]; iii) AAV-mediated gene therapy has also been exploited to express a TSP-1 fragment that inhibits human leukemia xenografts growth in nude mice [211]. iv) expression of 3TSR or of the second type I repeats containing the TGF-β-activating sequences significantly inhibited *in vivo* tumor angiogenesis and growth in nude mice [212]; v) expression of TSP-1-derived 4N1K peptide-containing proteins in renal cell carcinoma tissues was associated with a decrease in tumor growth and angiogenesis [213]; vi) transfection of a TSP-1 complementary cDNA antisense into glioblastoma cells lines significantly reduced TSP-1 production and cell motility [214]; vii) p53 inactivation lowered TSP-1 production [215,216]. Accordingly, topical delivery of p53 DNA to the lung increased TSP-1 expression, reduced microvessel density and limited lung tumor burden, prolonging the survival of tumor-bearing mice [217]; viii) c-Myc-regulated cluster miRNA-17-92, over-expressed in many human cancers, inhibited TSP-1 expression in cancer cells and in ECs. Inhibition of miR-17-92 by means of microRNAs increased TSP-1 expression and reduced VEGF-induced EC proliferation, migration and morphogenesis [218,219]; ix) transfection of c-Jun and/or RARalpha expression vectors into hepatoma cells and ECs raised mRNA and protein levels of TSP-1 [183]; x) over-expression of connexin-26 in human breast tumor cells up-regulated both the transcription and translation of TSP-1, retarding tumor growth *in vivo* [220]; xi) knockdown of Her-2/neu expression by siRNA increased the expression of TSP-1 and reduced that of VEGF [221]; xii) silencing CD26 using siRNA increased TSP-1 expression in T cells [200]. As discussed below, over-expression of TSP-1 or related molecules can sometimes lead to an increase in tumor growth, as after over-expression of the TSP-1 fragment 167-569 in C6 glioma cells [222].

### 4.3. TSP-1-based peptides and peptidomimetics

**Figure 7:**
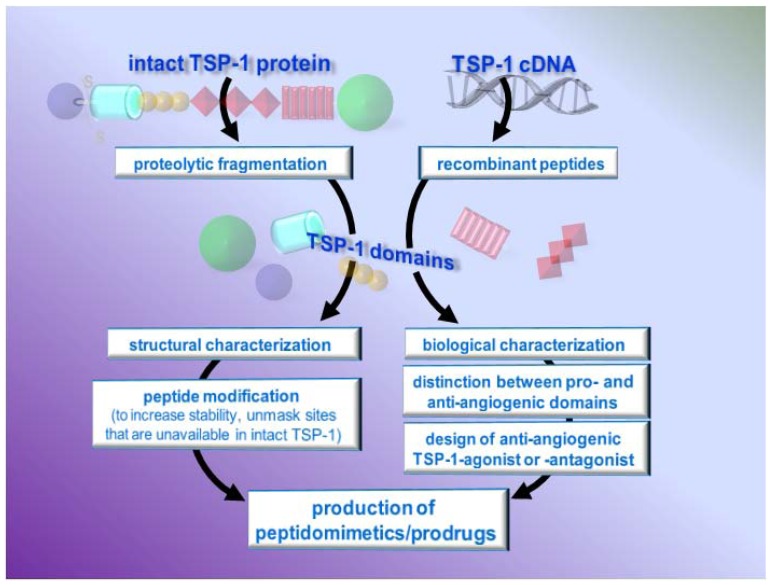
Design of anti-angiogenic TSP-1 peptides/peptidomimetics

The use of TSP-1-based drugs must deal with the fact that TSP-1, like its related peptides, can elicit both anti- and pro-angiogenic responses. However, this expands, rather than limits, the possibility of developing TSP-1-based antiangiogenic therapies by designing drugs or gene therapies that mimic the antiangiogenic effects of TSP-1 or antagonize the pro-angiogenic ones (Figure 7).

The use of biologically relevant, functional protein sequences as an entry point for the development of novel lead compounds is a powerful tool in drug discovery [223,224,225,226]. The starting sequences can elucidate the roles of key interactions (hot spots) in the regulation of important protein-protein interactions that a drug must agonize or antagonize. This knowledge may then boost our ability to interfere with specific pathological interactions, providing attractive therapeutic opportunities and extending medicinal chemistry to new classes of compounds.

#### 4.3.1. Characterization of TSP-1 active domains and sequences

The design of TSP-1-based drugs started from the identification/characterization of TSP-1 active domains. These studies used antibodies directed against the various portions of TSP-1 or peptides representing various TSP-1 fragments. The latter can be obtained by controlled proteolytic digestion of the intact TSP-1 protein [227] (e.g. by ADAMTS-1 [228] or thrombin [103]) or, more often, by the production of recombinant fragments [100]. Table 5 lists the TSP-1-derived peptides studied so far for their ability to regulate neovascularization. The pro-angiogenic domains of TSP-1 are mostly mapped in its N-terminal domain, while the main antiangiogenic sequences are in the second and third type I repeats.

Besides ECs, TSP-1-derived peptides also act on tumor cells. A peptide representing the Hep-I sequence induced promyelocytic leukemia cell differentiation and apoptosis [229], while 3TSR inhibited proliferation and induced apoptosis of B16F10 tumor cells in a TGF-β-dependent manner [114] *in vitro* and reduced tumor growth in orthotopic pancreatic xenografts [230,231] and in a model of polyoma middle T transgenic mice [232]. The peptide GGWSHW, located within the type I repeats, induces promyelocytic leukemia cell differentiation and apoptosis [229]. Retro-inverso peptides of the WSHWSxPWS sequence (aa sequence 438–452) inhibit the growth of MDA-MB-435 carcinoma cells in the mammary fat pad of nude mice [100,233]. WSxW-containing peptides also inhibit TSP-1-mediated activation of the TGF-β latent complex [133] and motility of glioma cells [214].

**Table 5:** Pro- and antiangiogenic TSP-1-derived peptides.

**Pro-angiogenic peptides**	**Mechanism**	**Reference**
peptides from the N-ter domain	increase proteolytic activity of EC interaction with integrins, HSPG, LRP disassembly of focal adhesion and EC migration	[78,103,129]
**antiangiogenic peptides**		
integrin–binding sequence of the N-ter domain	α_3_β_1_ integrin antagonists	[129]
sequences in the pro-collagen domain	various	[227]
various peptides from the second and third type I repeats	CD36-mediated EC apoptosis inhibition of EC response to AGF binding to protein/glycosaminoglycans TGF-βactivation integrin antagonist	[228,234,235]
peptide from the type III repeats	FGF-2 binding and sequestration	[8]
peptide 4N1 in the C-ter domain	CD47 binding	[122]

A fragment spanning the type III repeats and C-terminal domain causes promyelocytic leukemia NB4 cell death through CD47/α_ϖ_β_3_ [118]. Peptides containing the second type I repeats also inhibit tumor growth by regulating tumor cell proliferation and apoptosis in a TGF-β-dependent manner. Their capacity to inhibit angiogenesis is instead TGΦ−β−independent [134]. Finally, peptide 4N1, interacting with CD47, induces apoptosis in different breast cancer cell lines [236] and sensitizes human prostate tumor cells to taxane cytotoxicity [237], though it protects normal cells from apoptosis [238]. 

#### 4.3.2. Modifications of TSP-1-derived peptides and generation of peptidomimetics

The therapeutic exploitation of synthetic peptides is limited by their well-known shortcomings: unfavorable pharmacodynamics/pharmacokinetics (poor oral bioavailability and/or short duration of action), lack of receptor selectivity and low affinity (with K_d_ in the mM-μM range, compared to the pM-nM range of the K_d_ for parent proteins [239]). Modifying the peptide structure by acylation, PEGylation, fatty acid acylation, unnatural amino acids or restricted conformation can largely overcome these limits [240]. Many TSP-1 sequences exploitable for the design of antiangiogenic drugs are exposed in the TSP-1 molecule only after drastic structural changes induced by a low calcium concentration, different ligands, or reduction of disulfide bonds, indicating the structural modifications that must be introduced (and maintained) in the derived peptides. Guo and coworkers synthesized stereospecific analogs of the KRFKQDGGWSHWSPWSSC peptide from TSP-1 type I repeats that allowed dissection of different biological properties of the peptide enhancing the desiderable ones [233]. Similarly, the peptide D-reverse amKRFKQDGGWSH-WSPWSSac inhibits proliferation of C6 glioma cells *in vitro* and tumor growth *in vivo* [241]. Other modified TSP-1 peptides have been described: CVX-22 is a chimera obtained by fusing two mimetic nonamer peptides from TSP-1 type I repeats to the Fab binding site of a humanized scaffold antibody. This chimera selectively induces apoptosis of VEGFR-2-positive ECs in melanoma [242]. A phase I trial for this compound found some possibly drug-related adverse events but no dose-limiting toxicities [243].

ABT-510 is an antiangiogenic TSP-1 modified nonapeptide designed on the 7-mer active sequence GVITRIR of the second type I repeats. Although not active in the native conformation, appropriate modifications gave it strong antiangiogenic activity [75]. In detail, the first L-ile residue of the GVITRIR sequence was substituted with D-ile and the first Arg with the non-natural amino acid norvaline. The resulting D-enantiomer was capped by the addition of sarcosine at the N-terminus and proline ethylamide at the C-terminus, generating peptide ABT-526 [75]. A more soluble version of the peptide, eventually named ABT-510, was obtained by substituting D-ile with D-allo-ile [244,245]. Since its original preparation and description in 1999 [75], the antiangiogenic and antineoplastic activity of ABT-510 has been thoroughly investigated *in vitro*, *in vivo* and in humans, demonstrating that it has a favorable potency, solubility and pharmacodynamics/pharmacokinetics profile [245]. In ECs, it inhibits proliferation and migration, induces CD36-dependent apoptosis, and up-regulates CD95L/FasL. Besides acting on ECs, ABT-510 also induces apoptosis of CD36-expressing tumor cells [246], suggesting a double effect on both the vascular and tumor compartments, an important property of TSP-1 and related reagents that has been already mentioned above and that will be discussed further in this review. 

*In vivo* ABT-510 inhibits angiogenesis in different assays [244,245,247,248]. It reduces tumor growth and microvessel density in a ras-dependent/VEGF-independent tumor model [249], in syngeneic and xenograft gliomas [247], in orthotopic bladder cancer [244] and ovarian carcinoma xenografts [246] and in Lewis lung carcinoma [245]. Besides tumor growth, ABT-510 also inhibits metastasis in the B16F10 model [244] and ovarian cancer xenografts [246]. It has shown promising single-agent activity in canine cancer, inducing objective responses and disease stabilization [250].

On the basis of these favorable preclinical data, ABT-510 was tested, as a single agent, in three phase I and 4 phase II clinical trials between 2005 and 2008. Although phase I studies indicated that ABT-510 was safe and had a good toxicity profile even after several months’ use with different schedules [251,252,253], it showed little clinical activity on renal cell carcinoma, soft tissue sarcoma and melanoma [252,253,254,255]. These disappointing results, however, were no different from those already seen with other antiangiogenic agents used as single agents [253], justifying further evaluation in combination therapies [254,255]. Preclinical studies with the combination of ABT-510 and valproic acid [256] or CeeNu [250] gave favorable results and two phase I trials demonstrated the safety of ABT-510 in combination with chemotherapeutics such as gemcitabine, cisplatin and 5-FU/leucovorin [257,258].

A completely different and innovative rational approach that may overcome the limits of peptide-based antiangiogenic therapy is the identification of synthetic, non-peptidic molecules that mimic the hot-spot interactions in macromolecular complexes [224,259,260]. By using a peptide array approach followed by binding assays with synthetic peptides and recombinant proteins, we identified a FGF2 binding sequence of TSP-1 in the 15mer sequence DDDDDNDKIPDDRDN of the type III repeats. The peptide itself did not inhibit FGF2 function but served as a tool to identify the physico-chemical determinants of FGF2 recognition by TSP-1 and to design non-peptidic inhibitors. Nuclear magnetic resonance and molecular dynamics simulations taking into account the full flexibility of the ligand and receptor identified the relevant residues and conformational determinants for the peptide-FGF interaction. This information was translated into a pharmacophore model used to screen the NCI2003 small molecule databases, leading to the identification of three small molecules that bound FGF2 with affinities in the nanomolar range of concentration. These compounds prevented FGF2 binding to ECs, and inhibited FGF2-induced EC proliferation *in vitro*, and angiogenesis in the CAM assay. Although the lead compounds have still to be derivatized to improve the drug-like properties before they can be considered real drug candidates, our study show that it is feasible to develop small molecule mimics of TSP-1, and more in general of endogenous proteins, as therapeutic agents [8].

## 5. Conclusions

**Figure 8:**
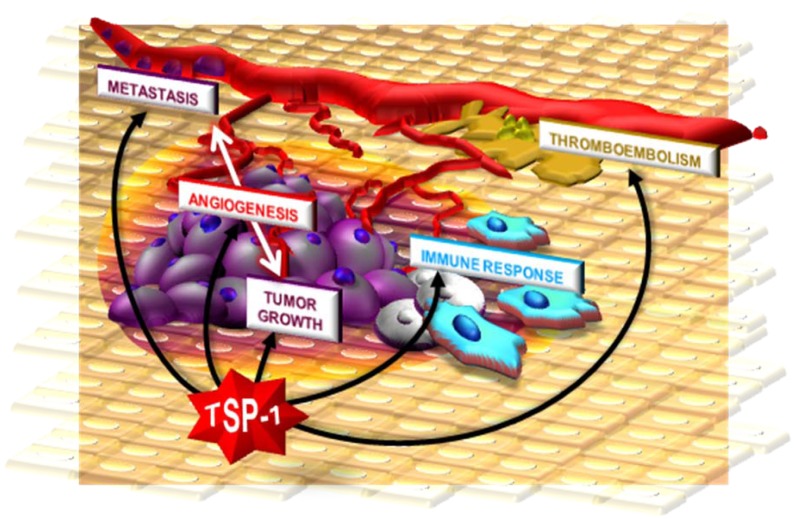
TSP-1 interferes with tumor progression at different levels. It blocks neovascularization, thus inhibiting tumor growth and metastasis which are further inhibited by its direct action on tumor cells. By acting on immune cells, TSP-1 may enhance the immune antineoplastic response. Finally, through its action on coagulation, TSP-1 may control the thromboembolic events that afflict oncological patients.

Among all the endogenous inhibitors of angiogenesis, TSP-1 seems the most promising for the development of efficacious antiangiogenic/antineoplastic therapies. TSP-1 can act with different mechanisms on different targets at cellular (leukocytes, endothelial, tumor and stromal cells) and molecular (AGFs, cell surface receptors, ECM) levels. Thus, TSP-1 can inhibit tumor progression not only through its well-known antiangiogenic action. It can induce an antineoplastic immune response by recruiting macrophages in the tumor and enhancing their cytotoxicity towards the tumor [261]. TSP-1 can also inhibit megakaryocytopoiesis [262] and coagulation [144,150,171], suggesting that its appropriate exploitation might help preventing the thromboembolic disorders that contribute to the morbidity/mortality of oncological patients [263]. Finally, and perhaps most importantly, TSP-1 can act directly on cancer cells, reducing their growth, inducing apoptosis [10,117,134,264,265,266], increasing their sensitivity to chemotherapeutics [237], and preventing metastatic dissemination [244,246]. Thus, TSP-1 can be positioned at the crossroads between tumor growth, angiogenesis, immunity and coagulation (Figure 8), extending its possibilities for therapeutic exploitation. 

However, TSP-1 can exert opposite effects on the immune response against tumors, as demonstrated by the fact that it inhibited TCR-mediated T lymphocyte early activation [267]. Also, peptide 4N1K induces cell death of monocytes and monocyte-derived DCs [268]. Paradigmatic of these divergent effects on the immune response is the observation that the absence of TSP-1 in “knock-out” mice can either increase [269] or attenuate [270] Th17 response. Also, while the TSP-1-N-terminal domain renders DCs phagocytic, the TSP-1-C-terminal domain causes a tolerizing phenotype in the same cells [271]. Thus, while the intact TSP-1 molecule inhibits phorbol myristate acetate/LPS-induced homotypic aggregation of human monocytes, the 70-kDa fragment of TSP-1 generated by proteolytic cleavage promotes homotypic aggregation [272]. Similarly, TSP-1 or its peptides can enhance thrombosis, instead of inhibiting it [150,273,274]. Finally, TSP-1 and the peptides can actually increase tumorigenicity [102,275,276,277,278] [192,222,279,280].

In conclusion, TSP-1 will remain an interesting source of therapeutic molecules for a variety of different applications, once the limits imposed by its structural and functional complexity are overcome by identification of the specific active sequence(s) and their proper exploitation.
